# Crystal structure, Hirshfeld surface analysis and energy framework calculation of the first oxoanion salt containing 1,3-cyclo­hexa­nebis(methyl­ammonium): [3-(aza­niumylmeth­yl)cyclo­hex­yl]methanaminium dinitrate

**DOI:** 10.1107/S2056989018008381

**Published:** 2018-06-12

**Authors:** Hammouda Chebbi, Samia Mezrigui, Meriam Ben Jomaa, Mohamed Faouzi Zid

**Affiliations:** a University of Tunis El Manar, Faculty of Sciences of Tunis, Laboratory of Materials, Crystal Chemistry and Applied Thermodynamics, 2092 El Manar II, Tunis, Tunisia; b University of Tunis, Preparatory Institute for Engineering Studies of Tunis, Street Jawaher Lel Nehru, 1089 Montfleury, Tunis, Tunisia

**Keywords:** oxoanion salt, crystal structure, organic dinitrate, Hirshfeld surface, fingerprint plots, energy framework

## Abstract

The title salt, C_8_H_20_N_2_^2+^·2NO_3_^−^, was obtained by a reaction between 1,3-cyclohexa­nebis(methyl­amine) and nitric acid. The cyclo­hexane ring of the organic cation is in a chair conformation with the methyl­ammonium substituents in the equatorial positions and the two terminal ammonium groups in a *trans* conformation. In the crystal, mixed cation–anion layers lying parallel to the (010) plane are formed through N—H⋯O hydrogen-bonding inter­actions; these layers are formed by infinite undulating chains running parallel to the [001] direction. The overall inter­molecular inter­actions involved in the structure were qu­anti­fied and fully described by Hirshfeld surface analysis. In addition, energy-framework calculations were used to analyse and visualize the three-dimensional topology of the crystal packing. The electrostatic energy framework is dominant over the dispersion energy framework.

## Abstract 2

In the organic cation of the title salt, the cyclo­hexane ring is in chair conformation with the two methyl­ammonium substituents in the equatorial positions. The crystal structure features extensive hydrogen-bonding inter­actions.

## Chemical context   

The design of new organic–inorganic hybrid ionic materials is of current inter­est for various applications, particularly in the areas of crystal engineering, supra­molecular chemistry and materials science (Kimizuka & Kunitake, 1996 ▸; Mitzi *et al.*, 1999 ▸; Bonhomme & Kanatzidis, 1998 ▸; Wachhold & Kanatzidis, 2000 ▸), and also for optical semiconductor materials (Kagan *et al.*, 1999 ▸; Li *et al.*, 2008 ▸). Among these hybrid compounds, organic nitrates are particularly inter­esting for their multiple applications including as catalytic precursors of numerous reactions, in biological treatment systems or as pharmaco­logical products (Brandán, 2012*a* ▸,*b* ▸, 2015 ▸; Castillo *et al.*, 2011 ▸; Torfgård & Ahlner, 1994 ▸).

1,3-Cyclo­hexa­nebis(methyl­amine) (CHMA) is used industrially as a hardener for ep­oxy resins, a raw material for the production of polyamides and iso­cyanates, a rubber chemical for paper-processing agents, in fiber treatment agents and in cleaning agents (Pham & Marks, 2012 ▸). It can also be used as an effective new cross-linking agent for the chemical modification of polyimide membranes (Shao *et al.*, 2005 ▸). We have previously reported on the use of the 1,3-cyclo­hexa­nebis(methyl­ammonium) dication in the syntheses of organic–inorganic hybrid ionic complexes (Huo *et al.*, 1992 ▸; Yang *et al.*, 2008 ▸), but to the best of our knowledge there are no reported salt forms containing an oxoanion and 1,3-cyclo­hexa­nebis(methyl­ammonium).

In a continuation of our recent studies of new hybrid compounds containing an organic cation and an inorganic oxoanion such as CrO_4_^2−^ (Chebbi *et al.*, 2000 ▸; Chebbi & Driss, 2001 ▸, 2002**a* ▸,b* ▸, 2004 ▸), Cr_2_O_7_^2−^ (Chebbi *et al.*, 2016 ▸; Ben Smail *et al.*, 2017 ▸), NO_3_^−^ (Chebbi *et al.*, 2014 ▸) and ClO_4_^−^ (Chebbi *et al.*, 2017 ▸; Ben Jomaa *et al.*, 2018 ▸), we report in this work the crystal structure, Hirshfeld surface analysis and energy-framework calculations for a new organic nitrate, (C_8_H_20_N_2_)[NO_3_]_2_ (I).[Chem scheme1] 
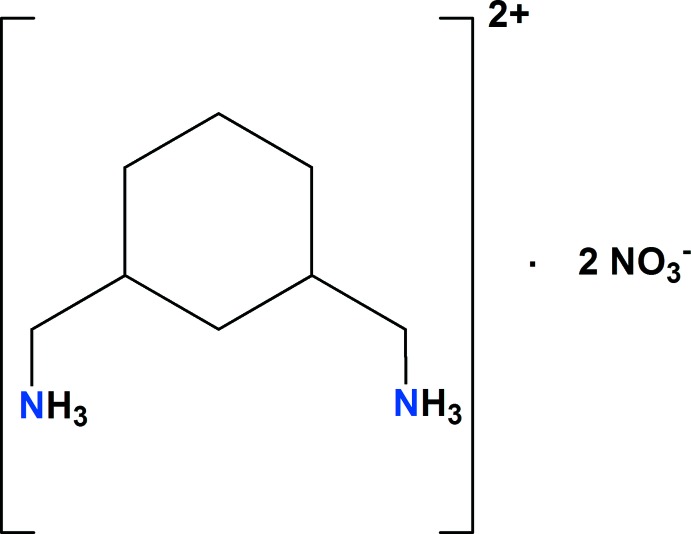


## Structural commentary   

**Figure 1:**
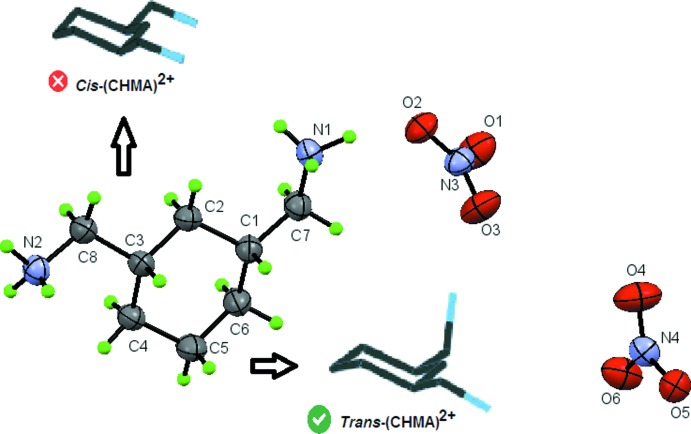
The asymmetric unit of (I), showing the atom-labeling scheme, displacement ellipsoids at the 30% probability level and the two configurations, cis and *trans*, of (CHMA)^2+^.

**Figure 2:**
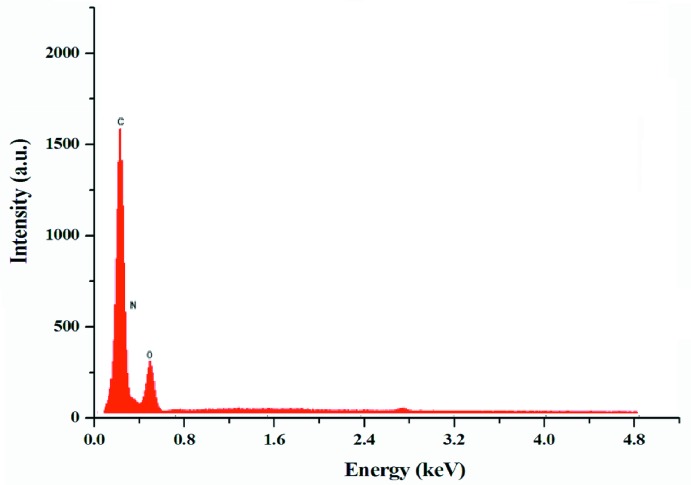
The EDX spectrum of (I), showing the presence of C, N, and O.

The title compound crystallizes with one 1,3-cyclo­hexa­ne­bis(methyl­ammonium) dication, (CHMA)^2+^, and two nitrate anions in the asymmetric unit (Fig. 1 ▸). An EDX spectrum confirming the presence of C, N, and O is shown in Fig. 2 ▸.

All atoms of the nitrate anion are coplanar with the N—O bond distances varying between 1.218 (4) and 1.250 (4) Å. The O—N—O angles are in the range of 118.8 (4)–121.1 (4)°. These bond lengths and angles are in good agreement with those observed in similar compounds (Blerk & Kruger, 2009 ▸; Gatfaoui *et al.*, 2017 ▸; Hakiri *et al.*, 2018 ▸). The cyclo­hexane ring of the organic cation adopts a chair conformation with the methyl­ammonium substituents in the equatorial positions and the two terminal ammonium groups in a *trans* conformation (Fig. 1 ▸). The same *trans* conformation has been observed in other compounds with this organic cation (Zhao *et al.*, 2008 ▸). Examination of the C—C (N) distances and C—C—C (N) angles in the (CHMA)^2+^ dication shows no significant difference from those in other organic materials associated with the same organic groups (Huo *et al.*, 1992 ▸; Yang *et al.*, 2008 ▸).

## Supra­molecular features   

**Figure 3:**
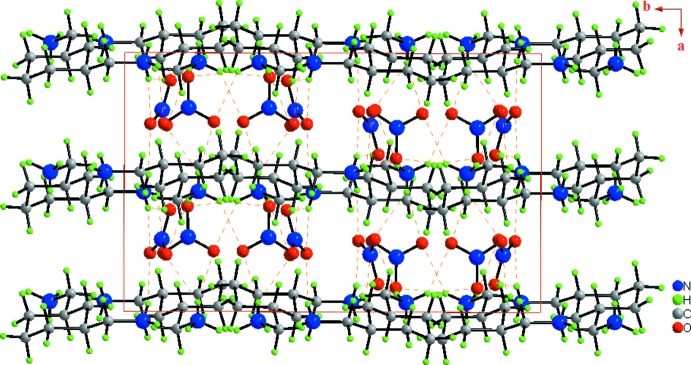
Structure of (I) viewed along the [001] direction, showing the infinite layers parallel to the *ac* plane. The N—H⋯O hydrogen bonds are shown as orange dashed lines.

**Figure 4:**
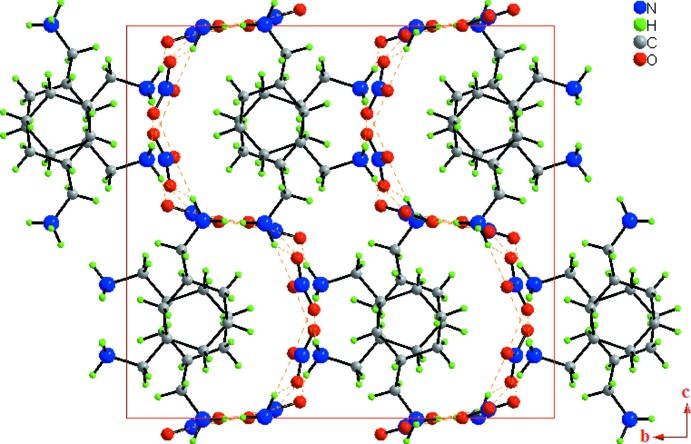
Unit-cell contents for (I) shown in projection down the *a* axis, showing the infinite undulating chains. The orange dotted lines indicate N—H⋯O hydrogen bonds.

**Figure 5:**
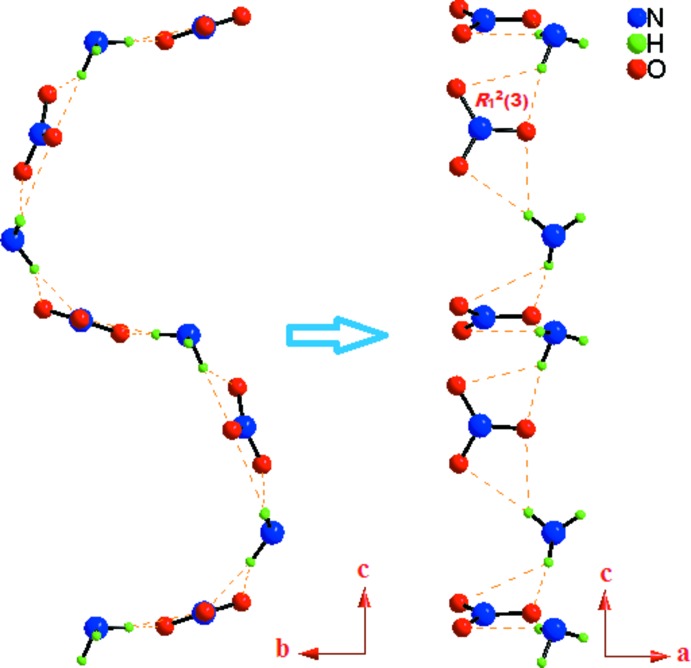
A view showing the 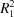
(3) motif built by N—H⋯O hydrogen bonds in the undulating chain. C and H atoms not involved in the inter­molecular inter­actions (dashed lines) have been omitted for clarity.

**Figure 6:**
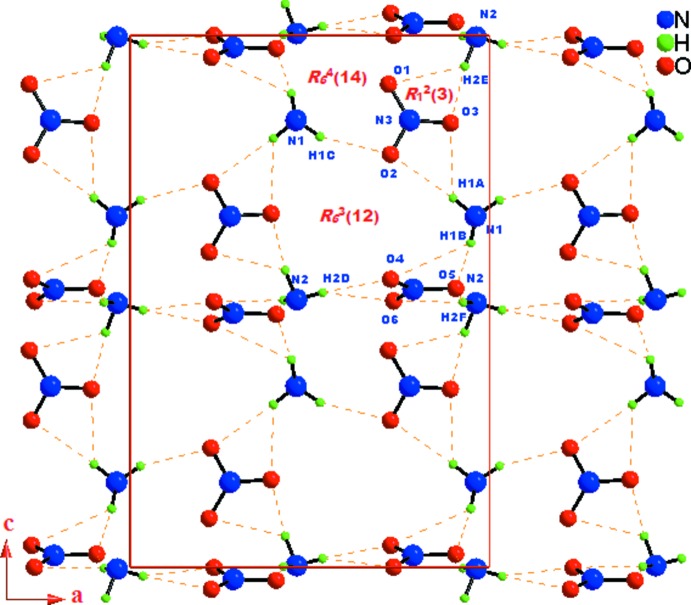
A view of the supra­molecular layer in the *ac* plane of (I), showing the formation of the 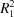
(3), *R_6_* ^3^(12) and *R_6_* ^4^(14) motifs. C and H atoms not involved in hydrogen bonds (orange dashed lines) have been omitted for clarity.

In the crystal, the (CHMA)^2+^ cations and nitrate anions are linked by N—H⋯O hydrogen bonds into infinite layers parallel to the *ac* plane (Fig. 3 ▸). Each layer is formed of infinite undulating chains running parallel to the [001] direction, further extended into an overall two-dimensional supra­molecular network structure (Fig. 4 ▸). As seen in Table 1 ▸, all of the hydrogen atoms bonded to the amine group of (CHMA)^2+^ dication contribute to the formation of N—H⋯O hydrogen bonds with the nitrate anions. The –N(1,2)H_3_^+^ groups of the organic cations, which act as donors to the N(3,4)O_3_^−^ anions generate 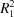
(3) dimeric rings. The propagation of these dimers produces infinite undulating chains running parallel to the [001] direction (Fig. 5 ▸). The inter­connection of two adjacent undulating chains leads to the generation of another two hexa­meric 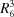
(12) and 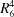
(14) ring motifs. Thus, the three types of 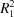
(3), 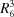
(12) and 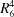
(14) rings are alternately linked into infinite layers parallel to the *ac* plane (Fig. 6 ▸).

## Hirshfeld surface analysis and energy framework calculations   

The Hirshfeld surfaces (Spackman & Jayatilaka, 2009 ▸) and their relative 2D fingerprint plots (Spackman & McKinnon, 2002 ▸; Parkin *et al.*, 2007 ▸; Rohl *et al.*, 2008 ▸)) were drawn using *CrystalExplorer 3.1* (Wolff *et al.*, 2012 ▸). The qu­anti­fying and decoding of the inter­molecular contacts in the crystal packing are visualized using *d*_norm_ (normalized contact distance) and 2D fingerprint plots, respectively. The dark-red spots on the *d*_norm_ surface arise as a result of short inter­atomic contacts, while the other inter­molecular inter­actions appear as light-red spots. *d*_i_ (inside) and *d*_e_ (outside) represent the distances to the Hirshfeld surface from the nuclei, with respect to the relative van der Waals radii. The proportional contribution of the contacts over the surface is visualized by the color gradient (blue to red) in the fingerprint plots.

**Figure 7:**
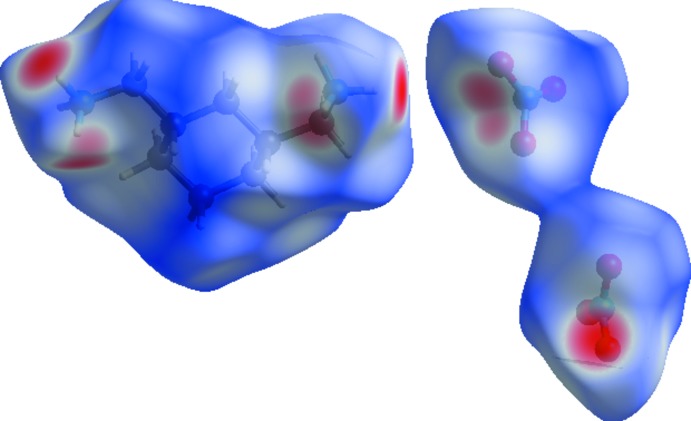
Hirshfeld surface around the constituents of (I) coloured according to *d*_norm_. The surface is shown as transparent to allow visualization of the orientation and conformation of the functional groups.

The Hirshfeld surface mapped over *d*_norm_ in the range 0.0620 to 0.9660 a.u. is illustrated in Fig. 7 ▸. Information regarding the inter­molecular inter­actions, visible as spots on the Hirshfeld surface (Fig. 7 ▸), is summarized in Table 1 ▸. For instance, the distinct circular depressions (red spots) are due to the N—H⋯O contacts, whereas the white spots are due to H⋯H contacts.

**Figure 8:**
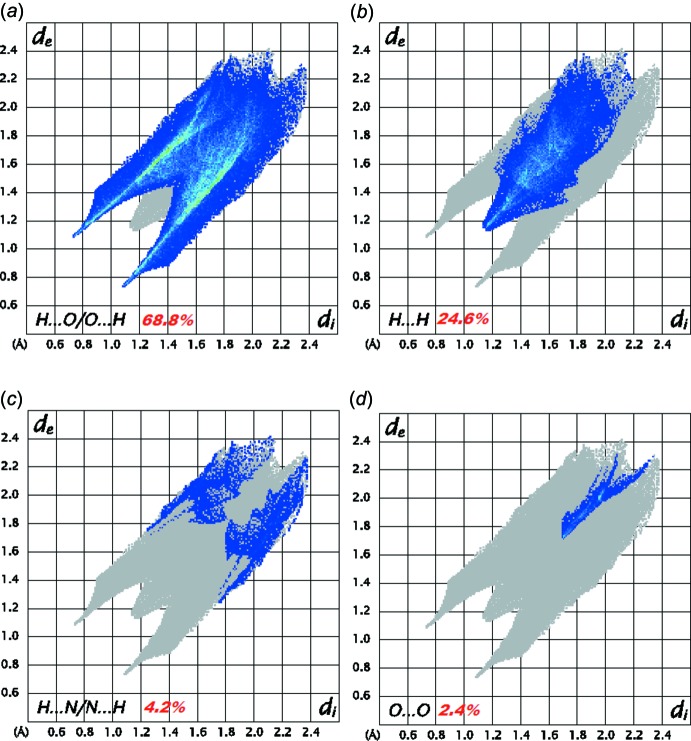
Two-dimensional fingerprint plots for (I) showing contributions from different contacts: (*a*) H⋯O/O⋯H, (*b*) H⋯H, (*c*) H⋯N/N⋯H and (*d*) O⋯O.

The inter­molecular inter­actions present in the structure are also visible on the two-dimensional fingerprint plot, which can be decomposed to qu­antify the individual contributions of each inter­molecular inter­action involved in the structure. The H⋯O/O⋯H contacts associated with N— H⋯O hydrogen bonding appear to be the major contributor to the crystal packing (68.8%); these contacts are represented by the spikes in the top-left (*d*_e_ > *d*_i_, H⋯O, 31.3%) and bottom-right (*d*_e_ < *d*_i_, O⋯H, 37.5%) regions of the related plots in Fig. 8 ▸ *a*. Inter­actions of the type H⋯H appear in the middle of the scattered points in the fingerprint plots; they comprise 24.6% of the entire surface (Fig. 8 ▸ *b*). The forceps-like tips in the region *d*_e_ + *d*_i_ ≃ 3 Å of the fingerprint plot (Fig. 8 ▸ *c*) represent a significant H⋯N/N⋯H contribution, covering 4.2% of the total Hirshfeld surface of (I). The O⋯O contacts, which represent only 2.4% of the Hirshfeld surface, Fig. 8 ▸ *d*, are extremely impoverished in the crystal (enrichment ratio *E*_OO_ = 0.17; Jelsch *et al.*, 2014 ▸), as the oxygen atoms bound to nitro­gen and the NO_3_ group as a whole are electronegative, therefore the O⋯O contacts are electrostatically repulsive.

**Figure 9:**
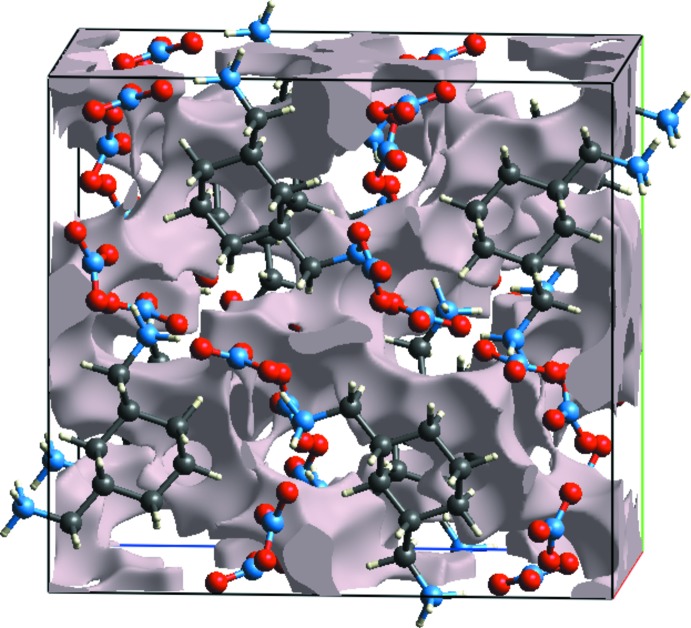
Void plot for (I).

Fig. 9 ▸ shows the voids (Wolff *et al.*, 2012 ▸) in the crystal structure of (I). These are based on the sum of spherical atomic electron densities at the appropriate nuclear positions (procrystal electron density). The crystal-void calculation (results under 0.002 a.u. isovalue) shows the void volume of title compound to be of the order of 469.14 Å^3^ and surface area in the order of 1334.82 Å^2^. With the porosity, the calculated void volume of (I) is 17%. There are no large cavities. We note that the electron-density isosurfaces are not completely closed around the components, but are open at those locations where inter­species approaches are found, *e.g*. N—H⋯O.

The crystallographic information file (crystal geometry and hydrogen bond distances to 1.083 Å) was used as input to *CrystalExplorer 17* (Turner *et al.*, 2017 ▸) and the inter­molecular inter­action energies were calculated for the energy-framework analysis. This calculation is estimated from a single-point mol­ecular wavefunction at B3LYP/6- 31G(*d,p*). A cluster of radius 3.8 Å was generated around the mol­ecule and the energy calculation was performed. The neighbouring mol­ecules (density matrices) are generated within this shell by applying crystallographic symmetry operations with respect to the central mol­ecule (density matrix). The inter­action energy is broken down as *E*_tot_ = *k*_ele_ *E′*_ele_ + *k*_pol_ *E′*_pol_ + *k*_dis_ *E′*_dis_ + *k*_rep_ *E′*_rep_ where the *k* values are scale factors, *E′*_ele_ represents the electrostatic component, *E′*_pol_ the polarization energy, *E′*_dis_ the dispersion energy, and *E′*_rep_ the exchange-repulsion energy (Turner *et al.*, 2014 ▸; Mackenzie *et al.*, 2017 ▸).

**Figure 10:**
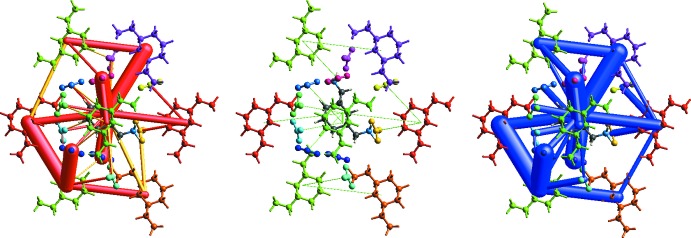
Energy framework diagram for separate electrostatic (left, red) and dispersion (middle, green) components of (I) and the total inter­action energy (right, blue). The energy factor scale is 60 and the cut-off is 5.00 kJ mol^−1^.

Table 2 ▸ shows the results of the inter­action energies calculations. The results are represented graphically in Fig. 10 ▸ as framework energy diagrams. The mol­ecular pair-wise inter­action energies calculated for the construction of energy frameworks are used to evaluate the net inter­action energies. The total inter­action energies are electrostatic (*E′*_ele_ = −247.8 kJ mol^−1^), polarization (*E′*_pol_ = −379.5 kJ mol^−1^), dispersion (*E′*_dis_ = −43.7 kJ mol^−1^), repulsion (*E′*_rep_ = 23.6 kJ mol^−1^), and total inter­action energy (*E*_tot_ = −566.3 kJ mol^−1^). The electrostatic energy framework is dominant over the dispersion energy framework (Fig. 10 ▸).

## Synthesis and crystallization   

1,3-Cyclo­hexa­nebis(methyl­amine) (1 mmol) was dissolved in water (10 mL) and nitric acid (2 mmol in 10 mL of water). The resulting solution was stirred for 3 h, filtered and then left to stand at room temperature. Colorless crystals were obtained after five days by slow evaporation.

## Refinement   

Crystal data, data collection and structure refinement details are summarized in Table 3 ▸. All C-bound hydrogen atoms were included in calculated positions with C—H = 0.98 (CH group) or 0.97 Å (methyl­ene) and allowed to ride, with *U*_iso_(H) = 1.2*U*_eq_(C). N-bound H atoms were located in difference-Fourier maps and freely refined.

## Supplementary Material

Crystal structure: contains datablock(s) I, global. DOI: 10.1107/S2056989018008381/xu5926sup1.cif

Structure factors: contains datablock(s) I. DOI: 10.1107/S2056989018008381/xu5926Isup2.hkl

Supporting information file. DOI: 10.1107/S2056989018008381/xu5926Isup3.cml

CCDC reference: 1847743

Additional supporting information:  crystallographic information; 3D view; checkCIF report

## Figures and Tables

**Table 1:** Hydrogen-bond geometry (Å, °)

*D*—H⋯*A*	*D*—H	H⋯*A*	*D*⋯*A*	*D*—H⋯*A*
N1—H1*A*⋯O2^i^	0.87 (4)	2.22 (4)	3.084 (6)	169 (4)
N1—H1*A*⋯O3^i^	0.87 (4)	2.36 (4)	3.013 (5)	132 (3)
N1—H1*B*⋯O4^ii^	0.95 (4)	2.59 (4)	3.187 (6)	121 (3)
N1—H1*B*⋯O5^ii^	0.95 (4)	1.87 (4)	2.818 (5)	172 (3)
N1—H1*C*⋯O2^iii^	0.87 (5)	2.12 (5)	2.939 (6)	157 (4)
N2—H2*D*⋯O4^iv^	0.77 (4)	2.48 (4)	3.142 (6)	145 (4)
N2—H2*D*⋯O6^iv^	0.77 (4)	2.19 (4)	2.899 (6)	153 (4)
N2—H2*E*⋯O1^iv^	0.99 (4)	2.46 (4)	3.178 (6)	130 (3)
N2—H2*E*⋯O3^iv^	0.99 (4)	1.88 (4)	2.858 (5)	173 (4)
N2—H2*F*⋯O5^v^	0.96 (6)	2.05 (6)	2.967 (5)	159 (5)
N2—H2*F*⋯O6^v^	0.96 (6)	2.23 (6)	3.016 (6)	139 (4)

Symmetry codes: (i) 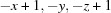; (ii) 
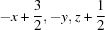
; (iii) 
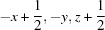
; (iv) 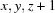; (v) 
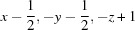
.

**Table 2:** Inter­action energies *N* refers to the number of mol­ecules with an *R* mol­ecular centroid-to-centroid distance (Å). Energies are in kJ mol^−1^.

*N*	Symop	*R*	*E*′_ele_	*E*′_pol_	*E*′_dis_	*E*′_rep_	*E*_tot_
2	−*x*, −*y* + 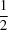 , *z* + 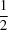	8.51	−63.9	−50.6	−6.3	5.4	−107.1
1	−*x*, −*y*, −*z*	9.69	32.1	−42.9	−4.6	1.8	−0.7
2	*x*, *y* + 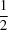 , −*z* + 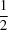	8.86	0.0	−53.8	0.0	0.0	−39.8
2	*x* + 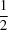 , −*y* + 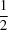 , *z*	5.33	−23.1	−75.4	−20.1	14.1	−89.0
1	*x* + 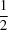 , −*y* + 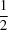 , *z*	5.60	−21.5	−50.6	−6.3	2.1	−64.4
1	*x* + 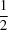 , −*y* + 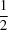 , *z*	5.77	11.3	−14.6	−0.5	0.0	0.7
1	*x* + 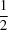 , −*y* + 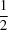 , *z*	5.33	−44.5	−18.9	−0.8	0.0	−61.8
1	−*x*, −*y*, −*z*	7.97	−128.4	−53.8	−4.3	0.2	−179.2
1	−*x*, −*y*, −*z*	6.43	−9.8	−18.9	−0.8	0.0	−25.0

Scale factors used to determine *E*_tot_: *k*_ele_ = 1.057, *k*_pol_ = 0.740, *k*_dis_ = 0.871, *k*_rep_ = 0.618 (Mackenzie *et al.*, 2017 ▸).

**Table 3:** Experimental details

Crystal data	
Chemical formula	C_8_H_20_N_2_^2+^·2NO_3_^−^
*M*_r_	268.28
Crystal system, space group	Orthorhombic, *P* *b* *c* *a*
Temperature (K)	293
*a*, *b*, *c* (Å)	10.475 (4), 16.884 (4), 15.514 (2)
*V* (Å^3^)	2743.8 (13)
*Z*	8
Radiation type	Mo *K*α
μ (mm^−1^)	0.11
Crystal size (mm)	0.52 × 0.40 × 0.15

Data collection	
Diffractometer	Enraf–Nonius CAD-4
Absorption correction	ψ scan (North *et al.*, 1968 ▸)
*T*_min_, *T*_max_	0.946, 1.000
No. of measured, independent and observed [*I* > 2σ(*I*)] reflections	3798, 2980, 991
*R*_int_	0.036
(sin θ/λ)_max_ (Å^−1^)	0.638

Refinement	
*R*[*F* ^2^ > 2σ(*F* ^2^)], *wR*(*F* ^2^), *S*	0.062, 0.173, 0.98
No. of reflections	2980
No. of parameters	187
H-atom treatment	H atoms treated by a mixture of independent and constrained refinement
Δρ_max_, Δρ_min_ (e Å^−3^)	0.17, −0.13

Computer programs: *CAD-4 EXPRESS* (Duisenberg, 1992 ▸; Macíček & Yordanov, 1992 ▸), *XCAD4* (Harms & Wocadlo, 1995 ▸), *SHELXS97* (Sheldrick, 2008 ▸), *SHELXL2014* (Sheldrick, 2015 ▸), *DIAMOND* (Brandenburg, 2006 ▸), *Mercury* (Macrae *et al.*, 2006 ▸), *WinGX* (Farrugia, 2012 ▸) and *publCIF* (Westrip, 2010 ▸).

## supplementary crystallographic information

### Crystal data

**Table:**

C_8_H_20_N_2_^2+^·2NO_3_^−^	*D*_x_ = 1.299 Mg m^−^^3^
*M**_r_* = 268.28	Mo *K*α radiation, λ = 0.71073 Å
Orthorhombic, *P**b**c**a*	Cell parameters from 25 reflections
*a* = 10.475 (4) Å	θ = 11–15°
*b* = 16.884 (4) Å	µ = 0.11 mm^−^^1^
*c* = 15.514 (2) Å	*T* = 293 K
*V* = 2743.8 (13) Å^3^	Plate, colorless
*Z* = 8	0.52 × 0.40 × 0.15 mm
*F*(000) = 1152	

### Data collection

**Table:**

Enraf–Nonius CAD-4 diffractometer	991 reflections with *I* > 2σ(*I*)
Radiation source: fine-focus sealed tube	*R*_int_ = 0.036
Graphite monochromator	θ_max_ = 27.0°, θ_min_ = 2.4°
ω/2θ scans	*h* = −13→2
Absorption correction: ψ scan (North *et al.*, 1968)	*k* = −1→19
*T*_min_ = 0.946, *T*_max_ = 1.000	*l* = −1→21
3798 measured reflections	2 standard reflections every 120 reflections
2980 independent reflections	intensity decay: 1%

### Refinement

**Table:**

Refinement on *F*^2^	0 restraints
Least-squares matrix: full	Hydrogen site location: mixed
*R*[*F*^2^ > 2σ(*F*^2^)] = 0.062	H atoms treated by a mixture of independent and constrained refinement
*wR*(*F*^2^) = 0.173	*w* = 1/[σ^2^(*F*_o_^2^) + (0.063*P*)^2^] where *P* = (*F*_o_^2^ + 2*F*_c_^2^)/3
*S* = 0.98	(Δ/σ)_max_ < 0.001
2980 reflections	Δρ_max_ = 0.17 e Å^−^^3^
187 parameters	Δρ_min_ = −0.13 e Å^−^^3^

### Special details

**Table:**

Geometry. All esds (except the esd in the dihedral angle between two l.s. planes) are estimated using the full covariance matrix. The cell esds are taken into account individually in the estimation of esds in distances, angles and torsion angles; correlations between esds in cell parameters are only used when they are defined by crystal symmetry. An approximate (isotropic) treatment of cell esds is used for estimating esds involving l.s. planes.
Refinement. Refinement of F^2^ against ALL reflections. The weighted R-factor wR and goodness of fit S are based on F^2^, conventional R-factors R are based on F, with F set to zero for negative F^2^. The threshold expression of F^2^ > 2sigma(F^2^) is used only for calculating R-factors(gt) etc. and is not relevant to the choice of reflections for refinement. R-factors based on F^2^ are statistically about twice as large as those based on F, and R- factors based on ALL data will be even larger.

### Fractional atomic coordinates and isotropic or equivalent isotropic displacement parameters (Å^2^)

**Table:**

	*x*	*y*	*z*	*U*_iso_*/*U*_eq_	
N1	0.5381 (5)	0.0463 (2)	0.6598 (3)	0.0659 (10)	
H1A	0.601 (4)	0.058 (2)	0.694 (3)	0.081 (16)*	
H1B	0.550 (3)	0.080 (2)	0.611 (2)	0.085 (13)*	
H1C	0.471 (4)	0.059 (3)	0.690 (3)	0.096 (19)*	
N2	0.4634 (5)	−0.1776 (3)	1.0032 (3)	0.0674 (10)	
H2D	0.534 (4)	−0.178 (3)	1.016 (3)	0.081 (19)*	
H2E	0.431 (4)	−0.155 (2)	1.057 (3)	0.098 (15)*	
H2F	0.426 (5)	−0.230 (3)	1.002 (3)	0.16 (2)*	
C1	0.5681 (4)	−0.0935 (2)	0.7032 (2)	0.0606 (10)	
H1	0.6549	−0.0819	0.7232	0.073*	
C2	0.4800 (4)	−0.0856 (2)	0.7804 (2)	0.0652 (11)	
H2A	0.4832	−0.0316	0.8016	0.078*	
H2B	0.3930	−0.0965	0.7626	0.078*	
C3	0.5166 (4)	−0.1416 (2)	0.8520 (2)	0.0601 (10)	
H3	0.6037	−0.1279	0.8695	0.072*	
C4	0.5197 (4)	−0.2269 (2)	0.8201 (2)	0.0652 (11)	
H4A	0.5512	−0.2610	0.8657	0.078*	
H4B	0.4338	−0.2437	0.8058	0.078*	
C5	0.6040 (4)	−0.2355 (2)	0.7419 (2)	0.0784 (12)	
H5A	0.6920	−0.2259	0.7583	0.094*	
H5B	0.5979	−0.2893	0.7204	0.094*	
C6	0.5673 (4)	−0.1791 (2)	0.6718 (2)	0.0741 (12)	
H6A	0.4826	−0.1923	0.6510	0.089*	
H6B	0.6264	−0.1845	0.6241	0.089*	
C7	0.5393 (4)	−0.0372 (2)	0.6311 (2)	0.0701 (11)	
H7A	0.6030	−0.0436	0.5862	0.084*	
H7B	0.4568	−0.0503	0.6066	0.084*	
C8	0.4321 (4)	−0.1278 (2)	0.9289 (2)	0.0710 (11)	
H8A	0.4386	−0.0727	0.9458	0.085*	
H8B	0.3442	−0.1377	0.9123	0.085*	
N3	0.2781 (4)	−0.09045 (18)	0.1598 (2)	0.0718 (9)	
O1	0.2195 (3)	−0.10116 (18)	0.0922 (2)	0.1032 (11)	
O2	0.2248 (3)	−0.06216 (17)	0.22373 (18)	0.0826 (9)	
O3	0.3922 (3)	−0.1097 (2)	0.16492 (19)	0.1031 (11)	
N4	0.7872 (4)	−0.1535 (2)	0.0224 (2)	0.0685 (9)	
O4	0.7259 (3)	−0.09475 (18)	0.0414 (3)	0.1171 (12)	
O5	0.9065 (3)	−0.15214 (15)	0.02450 (17)	0.0747 (8)	
O6	0.7335 (3)	−0.21487 (18)	0.0004 (2)	0.1000 (11)	

### Atomic displacement parameters (Å^2^)

**Table:**

	*U*^11^	*U*^22^	*U*^33^	*U*^12^	*U*^13^	*U*^23^
N1	0.070 (3)	0.063 (3)	0.065 (2)	−0.009 (2)	0.001 (3)	0.010 (2)
N2	0.071 (3)	0.078 (3)	0.054 (2)	0.000 (2)	0.001 (2)	0.0046 (19)
C1	0.064 (3)	0.059 (2)	0.058 (2)	−0.004 (2)	0.004 (2)	−0.001 (2)
C2	0.073 (3)	0.056 (2)	0.066 (2)	0.009 (2)	0.005 (2)	0.0013 (19)
C3	0.062 (2)	0.058 (2)	0.060 (2)	0.005 (2)	0.000 (2)	0.009 (2)
C4	0.072 (3)	0.054 (2)	0.069 (2)	0.003 (2)	0.000 (2)	0.0053 (19)
C5	0.098 (3)	0.058 (2)	0.080 (3)	0.013 (2)	0.011 (3)	−0.001 (2)
C6	0.091 (3)	0.062 (2)	0.070 (3)	0.003 (2)	0.016 (2)	0.000 (2)
C7	0.084 (3)	0.063 (3)	0.063 (2)	0.000 (2)	0.007 (2)	0.000 (2)
C8	0.076 (3)	0.071 (2)	0.066 (2)	0.012 (2)	0.007 (2)	0.007 (2)
N3	0.074 (3)	0.068 (2)	0.074 (3)	0.001 (2)	0.005 (2)	−0.0120 (19)
O1	0.092 (2)	0.140 (3)	0.078 (2)	0.009 (2)	−0.0104 (19)	−0.0334 (19)
O2	0.081 (2)	0.0903 (19)	0.0764 (18)	0.0065 (17)	0.0102 (17)	−0.0262 (16)
O3	0.074 (2)	0.140 (3)	0.096 (2)	0.028 (2)	−0.0052 (19)	−0.033 (2)
N4	0.070 (3)	0.075 (2)	0.060 (2)	−0.003 (2)	−0.016 (2)	0.0003 (19)
O4	0.091 (2)	0.095 (2)	0.165 (3)	0.024 (2)	−0.026 (2)	−0.042 (2)
O5	0.071 (2)	0.082 (2)	0.0709 (18)	−0.0077 (16)	−0.0064 (16)	−0.0090 (14)
O6	0.084 (2)	0.0728 (17)	0.143 (3)	−0.0103 (17)	−0.033 (2)	−0.0060 (18)

### Geometric parameters (Å, º)

**Table:**

N1—C7	1.479 (5)	C4—C5	1.507 (5)
N1—H1A	0.87 (4)	C4—H4A	0.9700
N1—H1B	0.95 (4)	C4—H4B	0.9700
N1—H1C	0.87 (5)	C5—C6	1.496 (5)
N2—C8	1.464 (5)	C5—H5A	0.9700
N2—H2D	0.77 (4)	C5—H5B	0.9700
N2—H2E	0.99 (4)	C6—H6A	0.9700
N2—H2F	0.96 (6)	C6—H6B	0.9700
C1—C7	1.499 (5)	C7—H7A	0.9700
C1—C2	1.518 (5)	C7—H7B	0.9700
C1—C6	1.524 (5)	C8—H8A	0.9700
C1—H1	0.9800	C8—H8B	0.9700
C2—C3	1.508 (4)	N3—O1	1.229 (4)
C2—H2A	0.9700	N3—O2	1.234 (4)
C2—H2B	0.9700	N3—O3	1.241 (4)
C3—C8	1.503 (5)	N4—O4	1.218 (4)
C3—C4	1.523 (5)	N4—O6	1.227 (4)
C3—H3	0.9800	N4—O5	1.250 (4)
			
C7—N1—H1A	113 (3)	C5—C4—H4B	109.3
C7—N1—H1B	109 (2)	C3—C4—H4B	109.3
H1A—N1—H1B	104 (3)	H4A—C4—H4B	108.0
C7—N1—H1C	114 (3)	C6—C5—C4	111.9 (3)
H1A—N1—H1C	103 (4)	C6—C5—H5A	109.2
H1B—N1—H1C	113 (4)	C4—C5—H5A	109.2
C8—N2—H2D	115 (3)	C6—C5—H5B	109.2
C8—N2—H2E	112 (2)	C4—C5—H5B	109.2
H2D—N2—H2E	96 (4)	H5A—C5—H5B	107.9
C8—N2—H2F	114 (3)	C5—C6—C1	111.7 (3)
H2D—N2—H2F	113 (5)	C5—C6—H6A	109.3
H2E—N2—H2F	104 (4)	C1—C6—H6A	109.3
C7—C1—C2	114.3 (3)	C5—C6—H6B	109.3
C7—C1—C6	111.2 (3)	C1—C6—H6B	109.3
C2—C1—C6	109.4 (3)	H6A—C6—H6B	107.9
C7—C1—H1	107.2	N1—C7—C1	112.4 (3)
C2—C1—H1	107.2	N1—C7—H7A	109.1
C6—C1—H1	107.2	C1—C7—H7A	109.1
C3—C2—C1	111.9 (3)	N1—C7—H7B	109.1
C3—C2—H2A	109.2	C1—C7—H7B	109.1
C1—C2—H2A	109.2	H7A—C7—H7B	107.9
C3—C2—H2B	109.2	N2—C8—C3	113.8 (3)
C1—C2—H2B	109.2	N2—C8—H8A	108.8
H2A—C2—H2B	107.9	C3—C8—H8A	108.8
C8—C3—C2	109.8 (3)	N2—C8—H8B	108.8
C8—C3—C4	114.6 (3)	C3—C8—H8B	108.8
C2—C3—C4	111.0 (3)	H8A—C8—H8B	107.7
C8—C3—H3	107.0	O1—N3—O2	121.1 (4)
C2—C3—H3	107.0	O1—N3—O3	119.8 (4)
C4—C3—H3	107.0	O2—N3—O3	119.1 (4)
C5—C4—C3	111.5 (3)	O4—N4—O6	120.9 (4)
C5—C4—H4A	109.3	O4—N4—O5	120.3 (4)
C3—C4—H4A	109.3	O6—N4—O5	118.8 (4)

### Hydrogen-bond geometry (Å, º)

**Table:**

*D*—H···*A*	*D*—H	H···*A*	*D*···*A*	*D*—H···*A*
N1—H1*A*···O2^i^	0.87 (4)	2.22 (4)	3.084 (6)	169 (4)
N1—H1*A*···O3^i^	0.87 (4)	2.36 (4)	3.013 (5)	132 (3)
N1—H1*B*···O4^ii^	0.95 (4)	2.59 (4)	3.187 (6)	121 (3)
N1—H1*B*···O5^ii^	0.95 (4)	1.87 (4)	2.818 (5)	172 (3)
N1—H1*C*···O2^iii^	0.87 (5)	2.12 (5)	2.939 (6)	157 (4)
N2—H2*D*···O4^iv^	0.77 (4)	2.48 (4)	3.142 (6)	145 (4)
N2—H2*D*···O6^iv^	0.77 (4)	2.19 (4)	2.899 (6)	153 (4)
N2—H2*E*···O1^iv^	0.99 (4)	2.46 (4)	3.178 (6)	130 (3)
N2—H2*E*···O3^iv^	0.99 (4)	1.88 (4)	2.858 (5)	173 (4)
N2—H2*F*···O5^v^	0.96 (6)	2.05 (6)	2.967 (5)	159 (5)
N2—H2*F*···O6^v^	0.96 (6)	2.23 (6)	3.016 (6)	139 (4)

Symmetry codes: (i) −*x*+1, −*y*, −*z*+1; (ii) −*x*+3/2, −*y*, *z*+1/2; (iii) −*x*+1/2, −*y*, *z*+1/2; (iv) *x*, *y*, *z*+1; (v) *x*−1/2, −*y*−1/2, −*z*+1.
